# Lead and Zinc Uptake and Toxicity in Maize and Their Management

**DOI:** 10.3390/plants11151922

**Published:** 2022-07-25

**Authors:** Tayebeh Abedi, Shahin Gavanji, Amin Mojiri

**Affiliations:** 1 Division of Integrated Sciences for Life, Graduate School of Integrated Sciences for Life, Hiroshima University, 1-3-1 Kagamiyama, Higashi-Hiroshima 739-8526, Japan; abedi820@gmail.com; 2 Department of Biotechnology, Faculty of Advanced Sciences and Technology, University of Isfahan, Isfahan 8174673441, Iran; shahin.gavanji2@gmail.com; 3 Department of Civil and Environmental Engineering, Graduate School of Advanced Science and Engineering, Hiroshima University, Higashi-Hiroshima 739-8527, Japan

**Keywords:** biochar, genes, lead, maize, proteins, zinc, ZIP

## Abstract

Soil contamination with heavy metals is a global problem, and these metals can reach the food chain through uptake by plants, endangering human health. Among the metal pollutants in soils, zinc (Zn) and lead (Pb) are common co-pollutants from anthropogenic activities. Thus, we sought to define the accumulation of Zn and Pb in agricultural soils and maize. Concentrations of Pb in agricultural soil (in Namibia) could reach 3015 mg/Kg, whereas concentrations of Zn in soil (in China) could reach 1140 mg/Kg. In addition, the maximum concentrations of Zn and Pb were 27,870 and 2020 mg/Kg in maize roots and 4180 and 6320 mg/Kg in shoots, respectively. Recent studies have shown that soil properties (such as organic matter content, pH, cation exchange capacity (CEC), texture, and clay content) can play important roles in the bioavailability of Zn and Pb. We also investigated some of the genes and proteins involved in the uptake and transport of Zn and Pb by maize. Among several amendment methods to reduce the bioavailability of Zn and Pb in soils, the use of biochar, bioremediation, and the application of gypsum and lime have been widely reported as effective methods for reducing the accumulation of metals in soils and plants.

## 1. Introduction

The sources of heavy metals include both natural processes and human activities. Over past decades, more and more heavy metals of anthropogenic origin have been discharged into the environment, most of which have increasingly accumulated to potentially harmful levels in soils [1]. In addition, several human activities (such as wastewater irrigation, pesticides, chemical fertilizers, urban wastes, and metal mining) have led to the accumulation and contamination of heavy metals in agricultural soils [2]. Therefore, the accumulation of these metals in agricultural soils has become a vital problem worldwide as they can transfer into the food chain and threaten human health [1]. Moreover, when the heavy metal accumulation in soil is excessive, it can lead to crop loss and environmental and ecological deterioration [3]. Among heavy metals, zinc (Zn) and lead (Pb) are common soil co-pollutants from anthropogenic activities, such as severe soil degradation, automobile emissions, mining, and others [4]. Pb is one of the most toxic and widely reported metals in farmlands. Shi et al. [5] stated that more than 800,000 t of Pb had been released into the environment globally over five decades, most of which has accumulated in soil. Pb accumulation in soils affects environmental health and can impact human health and food quality. Furthermore, Pb affects the diversity of the biological population in soils. Biochemical processes, including nutrient cycling and soil organic matter breakdown, have also been influenced by high concentrations of Pb [6]. Another widely reported metal in soils is Zn. While Zn is an essential nutrient for the growth and development of plants, Zn at high concentrations in soils may cause metabolic disorders, become phytotoxic, and lead to a threat to human health from the food chain [7].

Consequently, the uptake and toxicity of Pb and Zn in plants were considered in this study. In addition, cereal crops (such as maize) are the major dietary sources of metal accumulation (such as of Pb and Zn) in humans, and therefore, reducing the metal transfer from soil to grains is a key issue for the food safety [8].

Maize is one of the main cereals produced worldwide and represents a basic food crop in human alimentation [9]. Chen et al. [10] stated that the production of maize (*Zea mays* L.) surpasses that of either wheat or rice. Furthermore, Wang et al. [11] stated that maize is an important and common agricultural crop worldwide that has been applied in several studies about metal pollution. Zampieri et al. [12] expressed that the global production of maize is estimated to be more than 1 × 10^9^ t. Hence, from the perspective of evaluating the uptake of Pb and Zn by plants, it would be valuable to give attention to the toxicity and mode of action of Pb and Zn in maize. The main goal of the current review is to study how minimizing the concentration of heavy metals in maize (as one of the most important food crops in the world) can be helpful in reducing the risk of food chain contamination.

## 2. Zn and Pb Accumulation in Farmlands Worldwide

Zn is an essential micronutrient for plants, and several plant species have developed strategies for securing or maximizing the utilization of Zn [13]. Intensive fertilizer use, wastewater or sewage sludge, and agricultural and animal wastes can cause the accumulation of Zn in many agricultural soils [14]. Zn accumulation in soil can affect soil fertility with phytotoxicity, microbial biomass, and soil macronutrient shortage (such as of phosphorous) [15].

Pb is naturally occurring in soils but mostly accumulates through anthropogenic activities, such as atmospheric deposition, mining, and gasoline use. Furthermore, the addition of Pb to soils via herbicides/pesticides has been frequently reported in the past [14]. Nyiramigisha et al. [15] expressed that the accumulation of Pb in soil can cause abnormalities in the metabolic function of microorganisms, shortages of soil macronutrients (such as phosphorus), decreases in urease, invertase, catalase, and acid phosphatase activity, and interruptions in water balance, mineral nutrition, and enzyme activity.

As described by Leštan et al., there are four main reactions that control the fractionation of heavy metals in soil [16], including: (1) adsorption/desorption because of ion-exchange and the formation of complexes and chemical bounds; (2) precipitation, usually with anions such as carbonate, phosphate, and sulfate, and participating as hydroxides; (3) penetration into the crystal structure of minerals and isomorphic exchange with cations; and (4) biological immobilization and mobilization. Zunaidi et al. [17] stated that valence, the speciation and charge of metal ions, and soil properties (such as clay, redox potential, pH, and organic matter content) can influence the behavior of metals in contaminated soils.

The type of agricultural soil is one of the most important factors that can affect the fate of heavy metals and their transfer in soils. Li et al. [18] expressed that soil minerals are key components of solid soil matrices. Clay minerals are important active components of soils that meaningfully affect the fixation and migration of metals within soils. It has generally been reported that clay plays a vital role in the accumulation of heavy metals. The adsorption of heavy metals with clay constituents is one of the important processes that defines the mobility and bioavailability of heavy metals in environments [19]. Ou et al. [20] stated that clay minerals commonly decrease the fractions of bioavailable/extractable heavy metals in soil. Clay minerals frequently have small particle sizes and high specific surface areas and contribute to the quantity of electric charge. Moreover, clay can adsorb heavy metals over inner-sphere complexation reactions [21]. In addition, clay particles contain commonly negative charge, which is a vital factor affecting the sorption properties of soil [22]. Two main types of clay minerals, based on the arrangement of tetrahedral and octahedral sheets, include 1:1 and 2:1 [23]. The 2:1 clays have a much greater surface area than the 1:1 clays due to the existence of an internal surface area. The 2:1 clays also have a greater cation exchange capacity (CEC) than the nonexpanding types; thus, the 2:1 clays have a much greater propensity for immobilizing metal ions [22]. Many studies [23,24] have shown that Zn and Pb can be fixed by sorption onto specific clay minerals.

Soil pH is another important factor that has a vital effect on Zn and Pb dynamics in soil and their uptake by plants [25]. Zwolka et al. [25] stated that the acidic pH of soil can be considered as one of the most vital factors affecting the mobility of metals in soil and their absorption by plants. Adamczyk-Szabela et al. [26] reported that a significant decrease in the Zn content of plants was observed with increasing soil pH levels up to 10. This may be a result of the increased Zn adsorption to soil with a high pH as the adsorption capacity of a solid soil surface that is usually enhanced by an increasing pH-dependent negative charge, chemisorption on calcite, co-precipitation in ferric oxides, and the formation of hydrolyzed forms of Zn [27]. However, the adsorption of metals on soil colloids is decreased at very acidic pH levels due to the competition of metals cations with H^+^ in adsorbing to colloids [28]. Leštan et al. [16] stated that the adsorption reactions of Pb and Zn are vital in soil at pH 3 to 5 and pH 5 to 6.5, respectively. Complexation and precipitation reactions of both Zn and Pb are dominant at pH 6 to 7.

Soil organic matter (SOM) plays a vital role in the mobility and uptake of Zn and Pb in soil and plants. Commonly, the solid phase of SOM is associated with the retention, decreased mobility, and bioavailability of trace metals; however, cationic metals, which would ordinarily precipitate at certain pH values, are sometimes maintained in solution via complexation with soluble organics [29]. In one study, the extractability of Pb was shown to be low in organic matter-rich soil, and the retention of Pb by SOM can be explained by the formation of organic complexes [30]. Oudeh et al. [31] found that SOM provides binding sites for metals. In another study, SOM strongly inhibited the precipitation of Pb at an acidic pH (3 to 4) [32]. Rutkowska et al. [27] stated that SOM has a dual effect on the concentration of Zn is soil solution. SOM enhances the adsorption of Zn to a solid phase; thus, it can decrease the Zn concentration in soil. However, high SOM levels can generate high dissolved organic carbon content, which can help form Zn complexes and result in higher concentrations of Zn in soil solutions. The study by Rutkowska et al. [27] also showed that the Zn activity in soil can increase with an increase in dissolved organic matter (DOM). DOM is a complex mixture of various molecules and is generally defined as the organic matter that can pass through a 0.45 μm filter. DOM can strongly bind Pb and Zn and play a vital role in controlling these metals in soil [33]. 

Table 1 shows the concentrations of Pb and Zn in agricultural soils worldwide, demonstrating that the accumulation and pollution of these metals in agricultural soils can be considered as a global issue. The greatest Pb concentration (up to 3015 mg/Kg) was reported in Namibia, whereas the greatest Zn concentration (1140 mg/Kg) was detected in Guilin (China).

## 3. Uptake of Pb and Zn by Maize and Effects of Their Toxicity on Maize

As mentioned above, the uptake of metals by maize roots depends on the metals’ availability in the soil solution, and this is related to several factors, mainly soil pH, presence and quantity of hydrous ferric oxide, soil properties, types of clay, and other factors. The reported accumulations of Pb and Zn in different parts of maize are shown in Table 2. The maximum Pb levels reported in roots, shoots, and grains was 27,870, 4180 (in China), and 245 (in India) mg/Kg, respectively, whereas the maximum Zn levels reported in roots, shoots, and grains was 6320, 2020 (in China), and 39.17 (in India) mg/Kg, respectively. Toxic levels of heavy metals have been reported to affect normal plant functions, disrupting metabolic procedures by modifying the permeability and enzymatic activity of the cell membranes in maize. Moreover, metals negatively interact with vital cellular biomolecules (such as nuclear DNA and proteins, which results in an increase in reactive oxygen species (ROS)) and disrupt the essential metal functionality in biomolecules (such as enzymes or pigments). A high Zn concentration in soil has been found to decrease initial chlorophyll fluorescence [63]. Furthermore, Zn toxicity can cause a blockage of xylem elements and inhibition of photosynthesis through the change in electron transport and the capacity of rubisco to fix CO_2_ [64] or through the cellular debris [65]. Apart from that, Rout and Das [66] stated, in high concentrations of Zn (7.5 mM of zinc), root cortical cells were obviously damaged. Moreover, they stated that necrosis can occur in mesophyll cells at high concentrations of Zn. In a study [67], inhibition of growth was reported after five weeks in high concentrations of Zn (400–1600 mM). High concentrations of Zn can significantly reduce growth rate and biomass, and inhibit cell elongation and division [67]. In another study [68], growth of maize was notably reduced in Zn toxicity conditions. In addition, a higher concentration of Zn causes higher accumulation of Zn in grains [69]. Islam et al. [70] stated that Zn, in high concentrations, may interfere with chlorophyll synthesis, which causes reduced photosynthesis and inhibition of plant growth.

Pb toxicity reduces root and plant growth and causes chlorosis and the blackening of roots. Pb can inhibit photosynthesis and reduce mineral nutrition and enzyme activities [71]. Pb toxicity causes an inhibition of seed generation and seedling growth and a decrease in the percent and index of germination [72]. Furthermore, Pb can be harmful to the cell membrane, and it alters its permeability, causes a reaction of sulphydryl (-SH) groups with cations, and reacts with phosphate groups and active groups of ADP and ATP [71]. In a study [73] on corn, the seed germination, length of roots and shoots, dry weights of roots and shoots, and total protein content were reduced at high concentrations of Pb. Sofy et al. [74] stated that the toxicity of Pb can negatively affect plant metabolism; thus, inhibition of plant growth can be caused by high concentrations of Pb in soils.

**Table 2:** Pb and Zn reported in Maize.

Plant’s Parts	Pb (mg/Kg)	Zn (mg/Kg)	Remark	Area	References
Grains	-	22.8	Maize was irrigated with wastewater	Shandong, China	[75]
Shoots	4180	6320	The concentrations (mg/Kg) of Pb and Zn were 1000 and 500 in soil, respectively. J934 was the main strain.	YuanJiang dry-hot valley, China	[76]
Roots	27,870	2020			
Grains	0.04	27.32	-	Guangxi, China	[77]
Roots	3.63	NR *	-	Sichuan Agricultural University, China	[78]
Stems	28.0	NR			
Grains	245	2.54	-	Kanwar wetland, India	[79]
Grains	18.28	39.17	-	Punjab, India	[80]
Fodder	0.02–1.1	NR	-	Multan City (Pakistan)	[81]
Grains	0.34	46.1	Soil texture was loess	Poland	[82]
Straw	8.1	504.0			
Roots	140.0	1958.0			
Grains	NR	30.7	Soil irrigated with sewage sludge	Embrapa-CNPMA, Brazil	[83]
Leaves	NR	7.89			
Leaves	0.26	22.87	-	Near Ikhueniro dumpsite, Nigeria	[84]
Shoot	1.31	63.81			
Stems	1.05	40.94			
Roots	2.62	89.55			
Leaf	76.0	32.4	-	Aba Egbira, Nigeria	[85]
Stem	46.2	21.0			
Root	16.2	5.6			

* NR = Not Reported.

The uptake of Pb and Zn increases with an increase in the availability of Pb and Zn in the soil [86]. Plants are capable of the uptake of metals (such as Zn and Pb) primarily through the plant roots via passive absorption, and some specific proteins facilitate metal transport in movement across the membrane (Soliman et al., 2019). The root cell walls first bind metal ions from the soil, and then the metal ions are taken up across the plasma membrane. The uptake of metal ions occurs via the secondary transporters (such as channel proteins and/or H^+^-coupled carrier proteins) [87]. With an increase in heavy metals concentration, the transportation and accumulation of metals in shoots and leaves are increased. In addition, several genes and proteins are involved in transporting Zn and Pb in maize.

### 3.1. Involved Genes and Proteins

**Figure 1:**
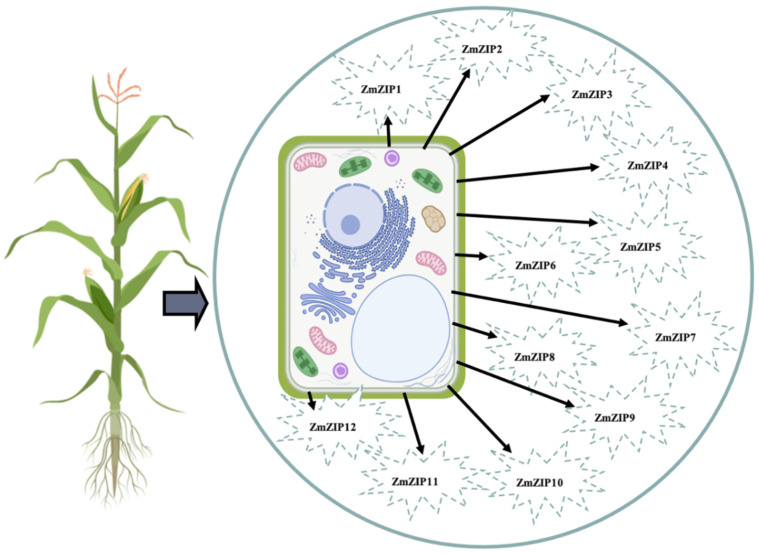
ZmZIPs are located throughout the plasma membranes of maize. This picture was created with BioRender.

The uptake and efflux of Zn are mainly controlled by different types of Zn transporters. Some of the central Zn transporters in the plants are Zrt-/Irt-like protein (ZIP family), plasma membrane-type ATPase (P-type ATPase family), heavy metal ATPase (HMAs family), yellow stripe-like (YSL), vacuolar iron transporter (VIT family), natural resistance-associated macrophage protein (NRAMP), and cation diffusion facilitators (CDFs) [88]. The Zrt-/Irt-like protein (ZIP) family can play significant roles in increasing and distributing Zn content in plant tissue [88,89]. ZIP (Figure 1) transporter proteins convey Zn^2+^ ions through the cell membrane [90]. Sequencing the maize genome has shown that there are 12 *ZIP* transporter genes (*ZmZIP1–ZmZIP12*), which are distributed on the 1, 3, 4, 6, and 8 chromosomes, these transporters are located in the plasma membrane in different parts of the plant. Mondal et al. [91] indicated that *ZmZIP* genes 1 to 11 are expressed in the flag leaf and 1 to 8 are also expressed in the root and shoot. They further stated that *ZmZIP3* and *ZmZIP12* are expressed in the kernel [92]. Multiple sequence alignment of ZmZIPs with ZIP transporter proteins in *Arabidopsis* (AtZIP), rice (OsZIP), and *Bordetella bronchiseptica* (BbZIP) revealed that metal-binding residues, including His177, Gly182, Glu211, and Gly212, are conserved in these plants [93]. Another study demonstrated that Glu141 and Glu170 act as Zn^2+^-coordinating residues and Met51, Ala47, His166, and Glu237 act as Zn^2+^-transporting residues in ZmZIP6, which are involved in Zn^2+^ binding and its transport. Based on that study, Glu282 and Asp190 in ZmZIP11 play a role as Zn^2+^-coordinating residues, and Met159, Ile74, Ala70, His278, and Lys198 are Zn^2+^-transporting residues [91]. Liu et al. [94] introduced novel Zn transporters in maize ZmLAZ1-4, which are located in the cell plasma membrane as well as chloroplast and vacuolar membranes They stated that the ZmLAZ1-4 located in plasma membrane plays a role in Zn uptake from the soil, and Zn transport into vacuole. However, the mechanism of ZmLAZ1-4 on the chloroplast is still unclear.

Numerous studies have been conducted to identify the responsive genes and proteins that are involved in Zn loading and accumulation in maize kernels. Quantitative trait locus (QTL) mapping can provide information that helps identify a gene’s activity in Zn accumulation [95,96]. The unique analysis of genomic resources, including single nucleotide polymorphisms (SNPs), insertion–deletion mutations (InDels), and differentially expressed genes (DEGs), provide information in recognizing genetic factors affecting Zn accumulation in maize. Interestingly, transcriptomic analysis of two different maize varieties with high and low Zn-containing kernels (VQL-2 and CM-145, respectively) showed that a greater number of transporters were up-regulated in VQL-2 than CM-145, which might lead to kernels with high Zn accumulation and Zn deficiency tolerance of the VQL-2 maize genotype. This study showed among 77 differentially expressed transporters belonging to five known families of Zn transporters (ZIP gene family, natural resistance-associated macrophage protein, P-type ATPase, and metallothionein family), P-type ATPase was the most abundant transporter [97]. Based on the results of research on heavy metal-associated isoprenylated plant proteins, *HIPPs* or *GRMZM2G104041,* which are located in the 4 chromosome, were found to encode heavy metal transport in maize (*Zea mays* L.) [98]. Cheng et al. [99] stated that the HIPP metallochaperones, including a metal binding domain, may have a vital role in heavy metal homeostasis and detoxification.

Identifying the genes and proteins that are involved in metal absorption and metal storage plays a key role in introducing metal-resistant plants and helps determine metal ion-binding proteins. To find Pb responses and tolerance mechanisms in maize (*Zea mays* L.), QTL analysis was used to identify the genetic basis of lead accumulation potential in maize [100] The RNA-seq data and qRT-PCR analysis showed that Pb concentrations in different maize tissues are determined by two genes associated with Pb, including *GRMZM2G137161* and *GRMZM2G132995*. These genes are located on the 2 and 6 chromosomes and they are significantly expressed during Pb stress [100]. Zhang et al. [101] analyzed the expression of transcription factors (TFs) under Pb stress in maize, and the results demonstrated that the overexpression of *ZmbZIP54* and *ZmbZIP107* can be improved considerably and can enhance Pb tolerance in maize. Two classes of heavy metal-binding proteins are metallothioneins (MTs) and phytochelatins (PCs), which are cysteine-rich and encoded by numerous genes [102]. A type 1 *MT* gene, which codes a protein with six N-terminal cys residues, was introduced into maize by de Framond [103], and in another study by Duan et al., a *ZmMT1*-encoded MT in maize was shown to be able to bind with Pb(II) and Zn(II) [104]. According to another study, the CNGC1 and CNGC2 proteins may be responsible for the transport of Pb^2+^ and potassium into plant tissue, and the uptake of Pb^2+^ occurs by calcium (Ca^2+^) and potassium (K^+^) channels in maize roots [105,106]. 

### 3.2. Phytosiderophores Mechanisms

Another mechanism, which may facilitate the uptake of metals (specially Fe) by the roots of maize, is the phytosiderophore. Phytosiderophores (PSs) are root exudates released by plants for the acquisition of Fe. Because of the high affinity of PSs for some other metals, they can solubilize micronutrients such as Zn [107]. PSs mostly occur where there is a deficiency of Zn and Fe [108]. One of the main PSs, which may release to increase uptake of Zn by plants, is the 2′-deoxymugineic acid [108]. In Zn–phytosiderophore mechanisms during the uptake of Zn from soils by maize, YS1 (ZMYS1), which belongs to a family of membrane transporters named YS1-like (YSL), plays a key role [109]. Some studies have stated that the PS does not have a significant effect on uptake of Cd and Pb [110].

## 4. Reducing the Uptake of Pb and Zn by Maize

The consumption of high concentrations of heavy metals by humans endangers human health and can cause several problems, such as headaches, gastrointestinal irritation, central nervous disorders accompanied by depression, extensive capillary damage, kidney damage, strong mucosal irritation, diarrhea, stomach cramps, vomiting, nausea, and liver damage [111]. To remove the heavy metals from soil and reduce their uptake by plants, several remediation methods comprised of physicochemical and biological methods have been used to recover land productivity [112]. Bioremediation, biochar amendment, and gypsum and lime amendment are widely used to reduce Pb and Zn uptake by plants.

### 4.1. Bioremediation

One of the frequently employed biological remediation techniques is the microbial immobilization, which has several advantages for environmental soundness, such as low hazardous material production and low energy consumption, and can stabilize metals in soil and limit their accumulation in plants [111]. Heavy metal-immobilizing bacteria have been described as having a strong immobilization capacity for metals (such as Pb and Zn) by biomineralization, the release of chelation agents, extracellular adsorption, and redox reactions [113]. Moreover, heavy metals can be adsorbed in ion form on the polysaccharides of bacteria by certain functional groups, such as amino, carboxyl, and sulfate groups [114]. Furthermore, some bacteria produce urease (enzyme) which can hydrolyze urea and increase soil pH and soil carbonate; therefore, soluble heavy metal ions in soil water can be converted to carbonate forms (mineralization) [115]. Cui et al. [116] stated that bacteria communities have a vital role in the absorption and inhibition of heavy metals by plants. In one study, *Actinobacteria* and *Proteobacteria* were the dominant bacteria able to reduce the uptake of metals by corn. *Actinobacteria* may primarily contribute in reducing the accumulation of Zn in corn, whereas *Proteobacteria* may primarily reduce the accumulation of Pb in corn [116]. In another study [113], two polyamine (PA)-producing strains, *Enterobacter bugandensis* XY1 and *Serratia marcescens* X43, were applied to reduce (by more than 52%) the uptake of Pb by a plant (spinach), and the main mechanism of Pb removal was via metal ion chelation by bacterially produced PAs, cell adsorption, and binding and precipitating on the bacterial cell surface in the form of PbO. *Rhodobacter sphaeroides* could reduce the exchangeable phase of Zn in soil by 100% [117]. On the basis of this study, *R. sphaeroides* may transform the available fractions of metals into less available and inert fractions, decreasing metal mobility and phytoavailability; however, it has been well accepted that the main mechanisms of bioremediation by *R. sphaeroides* are in metal sulfide formation [117]. A total of 98% of Zn and 90% of Pb were removed from soil by sulfate-reducing bacteria (*Desulfovibrio desulfuricans*), which can convert sulphate to hydrogen sulphate and subsequently react with metals to create insoluble forms [118]. Table 3 shows the reported bacteria for the bioremediation of Zn and Pb from contaminated soils.

Fungi and earthworms (Table 3) have also been widely used to remove Zn and Pb from contaminated soil. The main detoxification and removal of metals by fungi occurs through enzymatic processes, adsorption (biosorption) on extracellular structures (cell walls, capsules), extra- and intracellular precipitation, volatilization, and the chelation of metal/loids [136]. Some fungi (such as filamentous) usually accumulate metal ions into their mycelium and spores through the mechanisms that contain the fungal cell wall. In addition, some fungi (such as *T. ghanense*) also secrete ligninolytic enzymes that can increase the removal and biodegradability of heavy metals [137]. The removal of metals from contaminated soils by fungi can be conducted either with live fungi (bioremediation) or dead biomass (biosorption) [138]. Cell walls play a vital role in the sorption of metals [139]. A negatively charged cell wall surface plays a crucial role in the adsorption of metal ions via electrostatic attraction. As described by one study [136], the carbohydrate of the cell surface is comprised of various phosphodiester bridges in its side chains, which results in abundant negative charges on the surface of the cell. Phosphodiester bridges in both N- and O-linked mannosyl side chains are effective in metal binding via electrostatic attraction. In addition, fungal cell walls are frequently made up of polysaccharides, proteins, polyphosphates, lipids, polypeptides, chitin, and inorganic ions, which contain a number of functional groups such as -OH, -COOH, =NH, -SH, -NH_2_, -and O-CH_3_ and can bind with metals on the surface. In one study, more than 50% of Pb was removed with *Phanerochaete chrysosporium*, *Aspegillus awamori*, *Aspergillus flavus*, and *Trichoderma viride*. Furthermore, the majority of the fungal cells were able to tolerate up to 400 ppm metal concentrations [140].

Recently, vermiremediation, which is eliminating soil pollutants via earthworms, has been considered as a suitable and reliable method for soil remediation [141]. Earthworms can absorb toxic compounds from the soil through ingestion or the body wall, and they can enhance bioremediation and phytoremediation via improvement of microbial activity and plant growth [142]. The main mechanism of metal removal via worms is bioaccumulation. Earthworms can accumulate large amounts of metals in their gut tissue, such as in their chloragogenus tissue, which has a role as a cation exchange system for taking up heavy metals [142]. The rank order of bioaccumulation factor values for metal removal by earthworms (such as *Allolobophora rosea* and *Nicodrilus caliginosus*) were reported as Cd > Zn > Cu > As = Pb = Sb [142]. Moreover, earthworms have a protein (a MT) in their internal body that can bind with metals [143]. Zn (23.5–43.8%) has been removed by *Eisenia fetida* [144].

### 4.2. Biochar

Biochar is produced by the thermo-chemical conversion of biomass in a limited oxygen condition. Biochar has high porosity, which can adsorb metals and improve the growth of microorganisms to enhance biodegradation capability [145]. In addition, the application of biochar improves the properties of soils and fertility and immobilizes heavy metals in soils, reducing the uptake of metals by plants [146]. Kang et al. [147] stated that the application of biochar is a great way to remediate heavy metals from polluted soil. In addition, biochar applications can enhance the growth of plants. Biochar can decrease the availability of Zn and Pb in soils and reduce heavy metal uptake by plants [147]. In one study, the application of biochar was shown to decrease Zn by 21–28% in soil solution [148]. In several other studies [149,150,151], biochar applications have been shown to increase soil pH, which reduces the availability of metals, due to ash accretion and the dissolution of hydroxides and carbonates present in biochar [148]. Moreover, the high CEC, organic matter, and functional groups (such as: carboxylic acid (-COOH), -C=O-, and inorganic ionic (e.g., PO_4_)) of biochar may lead to the decreased availability and uptake of metals by plants [147]. Furthermore, the porosity and large surface areas of biochar lead to the adsorption of heavy metals and reductions in the concentrations of heavy metals in soils [152]. Table 4 shows the reduced concentrations of Zn and Pb in soil due to the application of biochar. The concentration of metals in the shoots of plants can be decreased with an application of biochar due to its ability to reduce the metal content in soil and roots. Up to 71% of Pb in shoots was reduced due to the application of biochar [153]. In another study [154], biochar amendments decreased Pb and Zn concentrations in shoots by 91% and 53%, respectively. As shown by Table 4, by amendment with biochar, the reduction in the availability of Pb is greater than the reduction in the availability of Zn.

### 4.3. Gypsum and Lime Amendment 

The application of gypsum and lime is another method, especially in acidic soils, to reduce the availability and mobility of Pb and Zn [162]. Kumpiene et al. [163] stated that gypsum and lime can effectively reduce the mobility of Cu and Pb in contaminated soils by raising soil pH. Dubrovina et al. [164] expressed that the application of gypsum (CaSO_4_ · 2H_2_O) seems to have an advantage in comparison with the application of lime as a soil amendment because of its high solubility (2.3–2.8 g L^−1^). In addition, it is generally applied as a subsoil acidity ameliorant [164]. In one study [165], using gypsum reduced 53% of Pb uptake by a plant (*A. gigas*). Combining with some amendment methods (including lime) reduced Zn (67.9%) and Pb (53.4%) uptake by shoots [166]. 

## 5. Conclusions

The accumulation of Pb and Zn by *Zea mays* L, as one important cereal, has attracted the attention of researchers. In the present study, many research papers were reviewed to study the journey of Pb and Zn from agricultural soils to maize. The key conclusions of this study are as below:

1. The maximum accumulation of Pb and Zn in soils was 3015 mg/Kg in Namibia, and 1140 mg/Kg in China.
2. The accumulation of Pb in the roots, shoots, and grains of maize reached 27,870, 4180, and 245 mg/Kg, respectively.
3. The accumulation of Zn in the roots, shoots, and grains of maize reached 2020, 6320, and 46.1 mg/Kg, respectively.
4. The Zrt-/Irt-like protein (ZIP) family can play a significant role in increasing and distributing Zn content in plant tissue.
5. The *GRMZM2G137161* and *GRMZM2G132995* genes are located on the 2 and 6 chromosomes, and they are significantly expressed during Pb stress.
6. Biochar, bioremediation, and amendment with gypsum and lime can play a great role in reducing the bioavailability of Pb and Zn in soils.
7. The genes involved in the uptake of Zn and Pb by maize have not been fully studied, which needs to be considered in future research.

## Figures and Tables

**Table 1:** Pb and Zn concentrations in soil globally.

Pb (mg/Kg, Average or Range)	Zn (mg/Kg)	Remarks	Area	References
30.7	85.8	Farmland	China	[34]
350.0	271.0	Agricultural soil	China	[35]
637.6	1140.0	NR *	Guilin, China	[36]
380.0	NR	Farmland	Northeast, China	[37]
32.4	76.0	Farmland	Taihang Piedmont Plain, China	[38]
31.2	NR	Farmland	Pearl River Delta, South China	[39]
33.0	92.6	Farmland	China	[40]
20.3–25.3	76.7–93.5	Agricultural soil	Siburan, Malaysia	[41]
600.0	NR	In dry seasons	Kangar, Malaysia	[42]
26.4	38.0	NR	Malaysia	[43]
18.2	38.3	Farmland	Allahabad, India	[44]
15.5	43.5	Farmland	Varansai, India	[45]
20.9–51.7	107.0–148.0	Agricultural land	Titagarh, India	[46]
254.6	117.0	Agricultural soil	Shiraz, Iran	[47]
0.67	NR	Farmland	Nigeria	[48]
0.53	0.40	Farmland	Near Shandam, Nigeria	[49]
304.5	206.6	Farmland (wet season)	Nigeria	[50]
12.8	28.0	Farmland	Kogi state, Nigeria	[51]
19–3015	27–104	Agricultural soil	Kombat mine, Namibia	[52]
12.9	40.6	NR	Inowroclawska Plain, Poland	[53]
18.9	35.8	NR	Romania	[54]
19.7	74.8	Agricultural land	Argolida basin, Greece	[55]
-	Up to 150	NR	European Union	[56]
3.64	52.2	Agricultural land	Turkey	[57]
13.0	35.0	Agriculture land	Uruçuí-Preto watershed, Brazil	[58]
11.2	16.2	Agricultural land	Pernambuco state, Brazil	[59]
15.2	41.2	Agricultural land	Argentina	[60]
29	23	Agricultural soil (top soil)	Sudbury, Canada	[61]
14	-	NR	Queensland, Australia	[62]
4.7	-	NR	Perth, Australia	[62]

* NR = Not reported.

**Table 3:** Bacteria, fungi, and earthworms for removal of Zn and Pb.

Species	Pb Removal (%)	Zn Removal (%)	Main Removal Mechanisms	References
Bacteria				
*Sporosarcina pasteurii*	33–85	21–66	Biomineralization	[119]
*Sporosarcina pasteurii* *Terrabacter tumescens* *UR53* *UR47* *UR41* *UR31*	88–99	88–99	Biomineralization	[115]
*Stenotrophomonas rhizophila* *Sporosarcina pasteurii* *Variovorax boronicumulans*	96.2 97.1 95.9	63.9 94.8 73.8	Biomineralization	[120]
*Bacillus brevis*	-	30–71	Biosorption	[121]
Cyanobacteria	-	96	-	[122]
*Bacillus* sp.	>50	-	Biosorption	[123]
*Bacillus* sp.	>60		Biomineralization	[124]
Fungi				
*Aspergillus niger*	40.8–45.5	-	Biosorption	[125]
*Pleurotus ostreatus ISS-1*	53.7	-	Extracellular biosorption, intracellular bioaccumulation, and precipitation with extracellular oxalic acids	[126]
*Aspergillus penicillioides*	>70	-	Bioaccumulation and biosorption	[127]
*Aspergillus flavus* *Sterigmatomyces halophilus*	- -	86 83	Biosorption	[128]
*Ascomycota*	-	36	Bioaccumulation Enzyme process	[129]
*Trichoderma brevicompactum* QYCD-6	97.5	4.6	Bioaccumulation	[130]
Earthworms				
*Eisenia fetida* and *Octolasion tyrtaeum*	58.4	25.0	Ingestion and bioaccumulation	[131]
*Eisenia fetida*	6–73	3–23	Ingestion and accumulation	[132]
*Eudrilus eugeniae*, *Eisenia fetida* and *Perionyx excavatus*	55.7	73.6	Bioaccumulation	[133]
*Lantana camara*	20	-	Bioaccumulation	[134]
*Libyodrillus violaceus*	3.5	18.5	Bioaccumulation	[135]

**Table 4:** Decreased availability of Zn and Pb in soil and maize due to amendment with biochar.

Soil/Roots/Shoots	Pb Removal (%)	Zn Removal (%)	References
Soil	50	54	[155]
	84–100	60–100	[151]
	-	19.8–35.6	[35]
	20.1–81.9	62.2	[156]
	-	57.0	[157]
	81.3–97.0	59.2–90.1	[158]
	-	76.4	[159]
	65.6	-	[160]
	12.8–34.6	-	[161]
Roots	55.0	-	[157]
	72.0	-	[160]
	-	22–57	[157]
	85.0	25.2	[146]
Shoots	-	40.0	[157]
	88.0	-	[160]
	-	40–67	[157]
	89.2	35.5	[146]

## Data Availability

The data presented in this study may be available on request from the corresponding author.
